# TGFβ‐mediated inhibition of hypodermal adipocyte progenitor differentiation promotes wound‐induced skin fibrosis

**DOI:** 10.1111/cpr.13722

**Published:** 2024-07-29

**Authors:** Meimei Yin, Lixiang Sun, Shuai Wu, Jinhang Ma, Wenlu Zhang, Xiaoxuan Ji, Zhichong Tang, Xiaowei Zhang, Yichun Yang, Xinyuan Zhang, Jin‐wen Huang, Shaoluan Zheng, Wen‐jie Liu, Chao Ji, Ling‐juan Zhang

**Affiliations:** ^1^ State Key Laboratory of Cellular Stress Biology, School of Pharmaceutical Sciences Xiamen University Xiamen China; ^2^ Central Laboratory, Zhongshan Hospital (Xiamen) Fudan University Xiamen China; ^3^ State Key Laboratory of Cellular Stress Biology, School of Life Sciences Xiamen University Xiamen China; ^4^ Department of Dermatology The First Affiliated Hospital of Fujian Medical University Fuzhou China; ^5^ Department of Plastic and Reconstructive Surgery, Zhongshan Hospital (Xiamen) Fudan University Xiamen China

## Abstract

Aberrant activation of dermal fibroblasts during wound healing often leads to debilitating fibrotic changes in the skin, such as scleroderma and keloids. However, the underlying cellular and molecular mechanisms remain elusive. Here, we established a wound‐induced skin fibrosis (WISF) mouse model in mature adult mice, characterised by excessive deposition of collagen bundles, loss of dermal adipocytes, and enrichment of DPP4^+^Ly6A^+^THY1^+^ hypodermal interstitial adipocyte progenitors (HI‐APs) and pericytes, resembling human fibrotic skin diseases. This WISF model exhibited an age‐dependent gain of fibrotic characteristics, contrasting with the wound‐induced hair neogenesis observed in younger mice. Through comprehensive analyses of the WISF, we delineated a trajectory of fibroblast differentiation that originates from HI‐APs. These progenitors highly expressed several extracellular matrix (ECM) genes and exhibited a TGFβ pathway signature. TGFβ was identified as the key signal to inhibit the adipogenic potential and maintain the fibrogenic potential of dermal APs. Additionally, administering a TGFβ receptor inhibitor to wound scar reduced the abundance of ECM‐producing APs. Finally, analysis of human scleroderma skin tissues revealed a negative correlation between the expression of AP‐, ECM‐, and TGFβ pathway‐related genes and PPARG. Overall, this study establishes a wound‐induced skin fibrosis mouse model and demonstrates that TGFβ‐mediated blockage of HI‐AP differentiation is crucial for driving fibrotic pathology. Targeting HI‐APs and adipogenesis may provide novel avenues for developing disease‐modifying therapies for fibrotic skin diseases.

## Graphical abstract

We establish a wound‐induced skin fibrosis (WISF) mouse model in adult mice, characterised by loss of dermal adipocytes and hair‐follicles and enrichment of hypodermal interstitial adipocyte progenitors (HI‐APs). HI‐APs are the origin of dermal fibrogenesis and main source of several ECM proteins, and TGFβ‐mediated blockage of HI‐AP differentiation drives WISF.

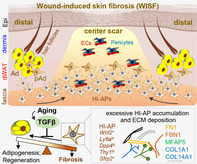

## 1 INTRODUCTION

Skin fibrosis is characterised by fibroblast proliferation, excessive extracellular matrix (ECM) deposition and the loss of specialised epithelial structures, which can cause debilitating and pathological changes in the skin, such as keloids, hypertrophic scars and scleroderma.^1^, ^2^, ^3^, ^4^, ^5^ Aberrant fibroblast activation during wound repair is a primary cause of skin fibrosis.^4^, ^6^, ^7^ However, fibroblasts are highly heterogeneous and plastic, and the origin of fibrotic fibroblast subtypes and the molecular and cellular mechanisms responsible for the dysregulation of fibroblast differentiation and the initiation of fibrosis are incompletely understood. Practical disease‐modifying therapies are lacking without a fully clarified pathogenesis; thus, the current therapies are primarily used to manage symptoms and relieve complications. Therefore, new insights into the pathological changes in fibroblasts during wound‐induced skin fibrosis are urgently needed to provide novel avenues for targeted therapy.

The skin, as one of the largest organs of the human body and a critical barrier protecting the human body against external insults, is a complex structure composed of several distinct layers, including the epidermis, papillary and reticular dermis and dermal white adipose tissue (dWAT).^8^, ^9^, ^10^ Recent studies have found that dWAT has a high degree of plasticity and can undergo drastic expansion or regression during skin development, normal hair cycling, and in response to external stimuli, such as wounding, inflammation or bacterial infection.^11^, ^12^, ^13^ Fibroblasts in the dWAT layer, called dermal adipocyte lineage cells,^14^ are highly heterogeneous. Recently, our team analysed PDGFRA^+^ dermal fibroblasts (dFBs) via single‐cell RNA sequencing (Sc‐RNAseq), validation sorting, imaging and functional analyses, identifying Ly6A^+^DPP4^−^ committed preadipocytes (pAd) and multipotent Ly6A^+^DPP4^+^ hypodermal interstitial adipocyte progenitors (HI‐APs).^14^

Recent studies have shown that cells of the hypodermal adipocyte lineage play critical roles in wound healing. Skin wound healing is a highly orchestrated process that includes an early haemostatic and inflammatory phase during the first few days, followed by a proliferative phase and a final remodelling phase that can take weeks to years.^15^, ^16^ During wound inflammation, Ly6A^+^DPP4^+^ HI‐APs migrate from the wound periphery and transdifferentiate into pAds or adipocytes, representing the majority stromal cell population in the newly formed wound granular tissue.^14^ Other studies have reported hypodermal/fascial DPP4^+^ dFBs (co‐expressing Cd201) as the primary fibroblast subtype that migrates from the peripheral hypodermis to the skin surface, dragging the surrounding ECM to form a provisional wound granular matrix.^17^, ^18^ Fascia fibroblasts are highly plastic. Our team found that sorted Ly6A^+^DPP4^+^ fascia fibroblasts are highly adipogenic and osteogenic in vitro, indicating they are multipotent progenitors.^14^ In vivo studies also suggest that fascia fibroblasts differentiate into pAd, adipocytes and myofibroblasts during wound healing.^14^, ^17^ However, how the dysregulation of dermal adipocyte lineage cells and their adipogenic differentiation programme affects the pathogenesis of wound‐induced skin fibrosis (WISF) remains elusive.

Therefore, this study explored the pathogenesis of WISF by first establishing a WISF mouse model followed by an in‐depth characterisation of the dFB subpopulation in the scar tissue to determine key pathways inhibiting the differentiation of APs into adipocytes. We aimed to provide insights into fibroblast heterogeneity and regulatory mechanisms in wound scars and offer a theoretical foundation for treating skin fibrosis by targeting dermal adipogenesis.

## 2 MATERIALS AND METHODS

**Animals and adult wound induced skin fibrosis mouse model:** C57BL/6 mice used in this study were bred and maintained in standard pathogen free (SPF) environment of the Laboratory Animal Center in Xiamen University. All animal experiments were approved by the Institutional Animal Care and Use Committee of Xiamen University. To generate large incisional skin wounds, 8 weeks old male C57BL/6 mice were anaesthetised and shaved, then full thickness excisional wounds (1.5 × 1.5 cm) were generated on the mid‐dorsal region of mouse skin. Mice were allowed to heal in the SPF animal facility, and pictures were taken every day to monitor wound healing. For TGFBR inhibitor administration, wounded mice were first allowed to heal upto 16 days then TGFBR inhibitor SB431542 (#S1067, Selleckchem) was injected intradermally every other day from day 16 to day 22 post wounding, and mice were sacrificed at day 24 post wounding for tissue collection and analyses.

**Human skin sample collection and analysis:** Adult human full thickness skin biopsies, from healthy, keloid and scleroderma donors, were collected by the department of dermatology, the First Affiliated Hospital of Fujian Medical University. All sample acquisitions were approved and regulated by Medical Ethics Committee of the First Affiliated Hospital of Fujian Medical University (reference number No. 2020[146]) or Medical Ethics Committee of the Zhongshan Hospital of Fudan University (reference number No. B2022‐626R). Upon collection, all samples were fixed with 4% PFA (#43368, Alfa Aesar) followed by paraffin embedding for histological or immunofluorescent analyses.

**Histology, collagen Masson staining and immunohistochemistry (IHC):** OCT‐embedded frozen mouse skin sections or paraffin‐embedded human skin sections were subjected to haematoxylin (#ZLI‐9610, ZSGB‐BIO) and eosin (#ZLI‐9613, ZSGB‐BIO) staining and/or masson's trichrome staining (#G1340‐7, Solarbio) according to manufacturer's protocol. Lipid staining was performed on frozen skin tissues by Bodipy (#D3922, ThermoFisher Scientific) dye, and actin fibres were counterstained with acti‐stain 555 conjugated phalloidin (PHA, #PHDH1‐A, Cytoskeleton) dye. For IHC staining, paraffin or OCT‐embedded sections were permeabilised with 0.1% saponin (#47036, Sigma), and blocked in 5% BSA (#4240GR100, Biofroxx) solution, then incubated with indicated primary antibodies (see Table S3 for antibody details) at 4°C overnight followed by appropriate fluorophore‐coupled secondary antibodies in the dark for 4–6 h at 4°C. Finally, mounting was performed by Biofix ProLong Gold Antifade Mountant with DAPI (Thermo Scientific Inc., Rockford, IL, US). After washing with PBS, the sections were mounted (#P36931, ThermoFisher Scientific) and observed by Leica TCS SP8 White Light Laser Confocal Microscope.

**Whole‐mount Oil Red O staining:** For whole mount Oil‐Red‐O (ORO) staining, freshly collected dorsal skin sample was pinned firmly onto a paraffin dish with underneath side up, and was fixed with 4% paraformaldehyde (PFA) for 20 min then loosely attached connective tissue was carefully removed by a scalpel under a dissection microscope. The skin sample was then washed with ddH_2_O and 60% isopropanol and incubated with Oil Red O Solution (0.3% Oil Red O in 60% isopropanol, #G1262, Solarbio) for 20 min. Unstained dyes were washed off by 50% isopropanol and ddH2O. Stained skin tissues were stored in ddH2O and pictures were taken by stereoscope Leica M165FC.

**Whole‐mount hair follicle neogenesis quantification:** To detect the alkaline phosphatase (ALP) in neogenic hair follicles in the wound centre, healed skin was collected at wound day (w.d.) 26 and incubated in 2 mg/mL dispase overnight at 4°C. Then, we gently peeled the epidermal portion off under a stereoscopic microscope (Leica S9), and the remaining dermis were fixed in 4% PFA for 10 min, rinsed thrice with PBS and incubated with BCIP/NBT (#C3206, Beyotime) to detect ALP for 30 min at 37°C. Lastly, the dermal preparations were rinsed twice with ddH2O to stop the reaction. Stained dermis were stored in ddH2O and pictures were taken.

**Fluorescence activated Cell Sorting (FACS) analysis:** FACS analysis was performed to analyse fibroblast heterogeneity in wound scar tissue as described previously.^11^, ^19^ Briefly, skin tissues were digested with collagenase D and DNase1 to isolate single‐cell suspension. Isolated skin cell suspension was stained with zombie violet viability dye (#423114, BioLegend), and blocked with CD16/CD32 Monoclonal Antibody (#14016185, ThermoFisher Scientific). Then cells were incubated with an antibody cocktail mix including PECy7 anti‐CD45 (#147704, BioLegend), PerCP‐Cy5.5 anti‐DPP4 (#2142977, ThermoFisher Scientific), PE anti‐THY1 (#105308, BioLegend), APC anti‐PDGFRa (#17140181, ThermoFisher Scientific) and BV605 anti‐Ly6A (#108133, BioLegend) antibodies for 1 h at 4°C. The stained cells were fixed and permeabilised with the fixation and permeabilisation buffer set (#88‐8824‐00, ThermoFisher Scientific), and stained with AF488 anti‐SMA antibody (#53976082, ThermoFisher Scientific) at room temperature for 30 min. After washing three times, the cells were resuspended with BD stabilising fixative buffer (#338036, BD biosciences). FACS analysis was performed by the Thermo Attune NxT machine and further analysed by FlowJo V10 software.

**Quantitative reverse transcription—PCR (qRT‐PCR) analyses and Bulk RNA sequencing:** Total cellular RNA was extracted from tissues or cultured cells by using Trizol (#T9424, Sigma), chloroform, and the RNAExpress Total RNA Kit (#M050, NCM Biotech), and 500 ng of total RNA was reversed transcribed to cDNA by using HiScript II Q RT SuperMix kit (#11123ES60, Vazyme). Quantitative PCRs were performed by SYBR Green qPCR Master Mix (#B21202, Bimake) on the Qtower real time machine (Analytikjena, Swavesey, Cambridge, UK). All primers used in our study were designed to span exon‐exon junctions, and *Tbp* (TATA‐Box Binding Protein) gene was used as a housekeeping gene to normalise target gene expression. Specific primer sequences are shown in Table S4. For bulk RNA sequencing, the quality of extracted/ purified total RNA was analysed by bioanalyzer (RIN value >7), and used to construct sequencing library according to the manufacturer's protocol (NEBNext Ultra RNA Library Prep Kit for Illumina). Then, the libraries were loaded on an Illumina Navoseq instrument for sequencing as described previously.^14^, ^19^ To classify and visualise the difference of biological processes in different groups, we present an R package ‘ClusterGVis’(v0.1.1) (https://github.com/junjunlab/ClusterGVis) to automate the process of DEGs clustering and the enrichment analysis of gene clusters in three groups. The gene ontology (GO) pathway analysis for differentially expressed genes was performed by R package clusterProfile 3.12.0. The bulk RNA‐seq dataset of skin biopsies samples from healthy controls and SSc patients shown in Figure 8 was downloaded from GSE130955^20^ and processed by R Program. Gene expression violin‐plots for single genes and expression correlation plots for two genes were generated and analysed by GraphPad Prism 10 software.

**Single cell RNA‐sequencing data analysis:** The single‐cell RNA‐seq (Sc‐RNAseq) dataset of the centre wound scar tissue was downloaded from GSE190175. Unsupervised clustering analysis was performed using Seurat 4.4.0.^21^ Cells with (1) greater than 20% mitochondrial RNA content, (2) less than 1000 genes detected were excluded from analysis. The preprocessed data were normalised and scaled using Seurat function NormalizeData, and FindVariableGenes was used to identify highly variable genes. The principal components (PCs) were estimated by RunPCA. t‐SNE dimensionality reduction was then performed by the RunTSNE to place cells with similar local neighbourhoods in dimension 1 to dimension 15 and visualise the distribution of cells. Clusters were identified by the FindNeighbors function on the basis of the top 15 significant principal components and the Findclusters function with resolution = 0.5. Gene tSNE and violin plots were plotted by the Dimplot and Vlnplot function. To evaluate the potential functionality of a cluster of interest, the AddMoudleScore function in the Seurat was used to calculate the functional signature scores of the cell cluster on the single‐cell level. For the trajectory analysis of dermal fibroblasts, branches and states were detected using the Monocle 2.26.0^22^ and arrows were detected using the Slingshot 2.10.0.^23^ In the monocle analysis, genes that define the trajectory were selected using the differentialGeneTest function and with *q*‐values <0.05. Dimensional reduction was performed using the DDRTree method and states were identified using the orderCells function. In the Slingshot analysis, the Seurat object with cluster information was converted to the SingleCellExperiment object, after which the “Slingshot” function was used to draw arrow to infer cell lineage and pseudotime. CytoTRACE 0.3.3^24^ with default parameter was used to predict differentiation states from scRNA‐seq data based on the simple yet robust observation that transcriptional diversity decreases during differentiation.

**Isolation, culture and adipocyte differentiation of primary mouse adipocyte progenitors:** Primary mouse dermal fibroblasts (dFBs) were isolated from neonatal mouse skin as described previously.^14^, ^19^ In brief, neonatal mouse skin samples were cut into small pieces and digested with 2.5 mg/mL Collagenase D (#10269638001, Sigma) and 1 mg/mL DNase1 (#D8071, Solarbio) for 2 h at 37°C. Then cell mixture was filtered with 40 μm filter, and incubated with red blood cell lysis buffer (#RT122, TianGen). Isolated neonatal dermal fibroblasts were cultured with DMEM medium (#C11965500BT, ThermoFisher Scientific) supplemented with 10% FBS in a humidified incubator at 5% CO_2_ and 37°C. Passage 1 cells were seeded to 24 well plate and cultured to post‐confluency prior to in vitro adipocyte differentiation by switching to adipocyte differentiation medium containing 2 μM Dexamethasone (#D4902, Sigma), 250 μM IBMX (#I5879, Sigma), 200 μM Indomethacin (#I8280, Sigma) and 10 μg/mL recombinant human insulin (#91077C, Sigma).

**Statistical analysis:** Experiments were repeated at least 3 times with similar results and were statistically analysed by GraphPad Prism 10 software. For experiments with two groups, statistical significance was determined using Student's unpaired two‐tailed *t*‐test. Normality was tested using the Shapiro–Wilk test, and for datasets that were not normally distributed, nonparametric tests were used to determine statistical significance. For experiments with more than two groups, one‐way ANOVA multiple comparison test was performed as indicated in the legend. All error bars indicate mean ± SEM. A *p* value of <0.05 was considered statistically significant (*p < 0.05; **p < 0.01; ***p < 0.001; ****p < 0.0001).

## 3 RESULTS

### 3.1 Establishing a WISF model in mature adult mice

**FIGURE 1:**
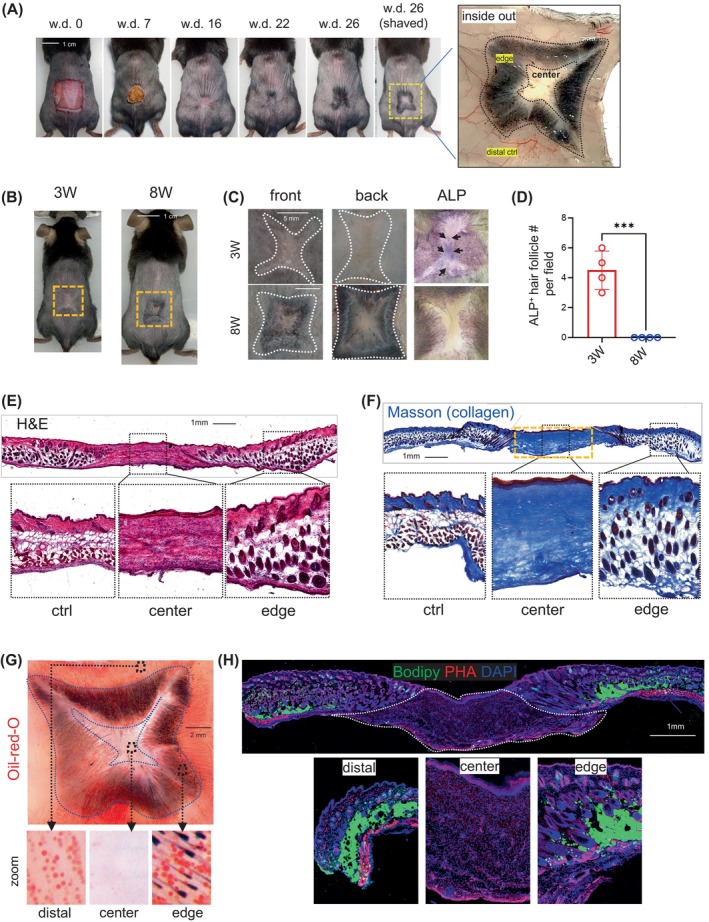
Establishment of a wound‐induced skin fibrosis model in mature adult mice. (A) Full thickness excisional dorsal wounds (1.5  × 1.5 cm) were created on mouse back skin and pictures were taken at indicated time points post wounding. The right panel shows the zoom‐in image of the undersurface (right) side of the wound at w.d. 26. (B–D) Mice at 3 weeks (3 W, young adult) or 8 weeks (8 W, mature adult) of age were subjected to large skin wounding model as described in A, and wounds were collected wound day (w.d.) 26 for imaging (B, C) and alkaline phosphatase staining (ALP in C). (D) Qualificative bar graphs showing the number of ALP+ hair follicles per field/wound (*n* = 4/group). All error bars indicate mean ± SEM. ***p < 0.001. (E–H) Skin wounds from mature adult mice w.d. 26 were subjected to various staining analyses, and zoom‐in images of the wound centre, edge or distal regions were shown in the lower panel. (E) HE staining showing skin histology. (F) masson staining showing collagen (blue) expression and deposition. (G) Whole‐mount Oil‐red‐O staining showing the distribution of ORO+ (red) lipid‐droplets. Note that hair follicles can be clearly visualised by black pigmentation. (H) Bodipy (green, marks lipid), PHA (red, phalloidin, marks actin fibre) and DAPI (blue) staining showing the distribution of bodipy^+^ (green) lipid‐droplets across skin section.

Chronic dysregulation of tissue repair and remodelling is a fundamental driver of skin fibrosis.^25^ We created a large excisional wound on the shaved back skin of adult C57BL/6 mice (8 weeks old) and allowed the wound to heal for up to 26 days (Figure 1A). During wound healing, hair neogenesis, indicated by black pigmentation, occurred around the peripheral (edge) region of the healed wound but not in the wound centre scar tissue (Figure 1A).

Although we did not observe hair follicle regeneration in the centre scar tissue of wounded adult mice, wound‐induced hair follicle neogenesis (WIHN) has been observed in younger mice when wounds are generated at 3 weeks of age.^26^, ^27^, ^28^, ^29^, ^30^ Therefore, to confirm age‐dependent changes in the ability of healing wounds to regenerate hair follicles, we created large excisional wounds in young adult mice (3 weeks old) and mature adult mice (8 weeks old) (Figure 1B). Alkaline phosphatase staining, which stains actively growing hair follicles,^31^ showed newly generated hair follicles in the centre scar tissue of the 3‐week‐old but not 8‐week‐old mice (Figure 1C,D). Histological (Figure 1E) and collagen staining (Figure 1F) of skin wound tissue from adult mice showed that the central scar tissue was devoid of skin appendages, including hair follicles, sebaceous glands and dermal adipocytes, but was packed with dense, thick, linearised collagen bundles, a phenotype that resembles those that define fibrotic skin diseases such as keloids, hypertrophic scars, and scleroderma.^32^, ^33^, ^34^ Therefore, to distinguish this model from the WIHN model, we termed this model the wound‐induced skin fibrosis (WISF) mouse model.

To further confirm the lipodystrophic phenotype, Oil‐Red‐O (ORO) whole‐mount and BODIPY staining were performed to detect the skin lipid content. ORO^+^ or BODIPY^+^ adipocytes were strikingly lost in the central scar tissue compared with the distal control or scar edge tissues (Figure 1G,H). Together we established a WISF model in mature adult mice characterised by extensive collagen deposition and a lack of skin appendages and dermal adipocytes in the central scar tissue.

### 3.2 PDGFRA ^+^ Ly6A ^+^ DPP4 ^+^ hypodermal APs and PDGFRA ^−^ ACTA2 ^+^ pericytes are enriched in the wound centre of the WISF model

**FIGURE 2:**
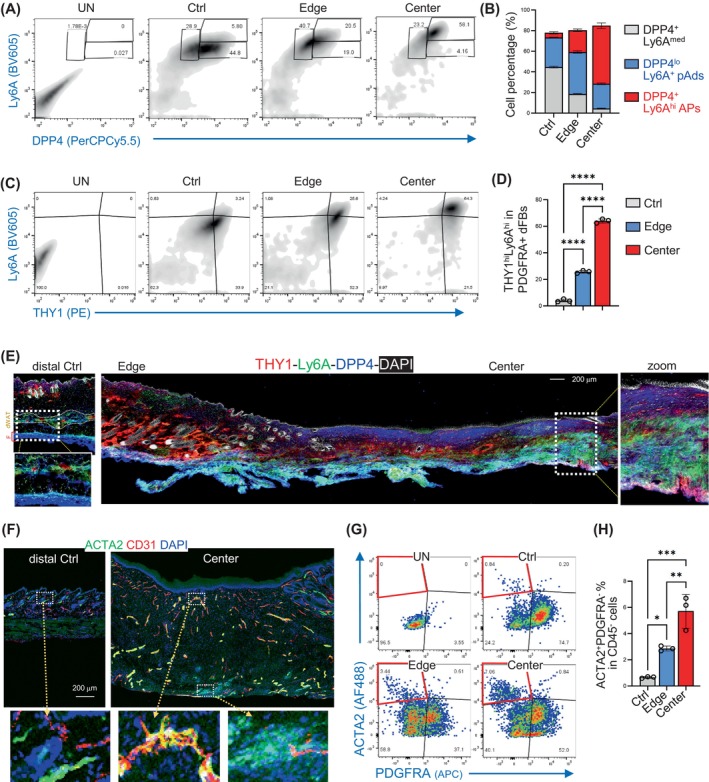
PDGFRA^hi^Ly6A^+^DPP4^+^THY1^hi^ hypodermal APs and PDGFRA^−^ACTA2^+^ pericytes are enriched in the wound centre of the WISF model. (A, B). Cells isolated from the distal control, edge or centre skin tissues from w.d. 26 wounds were subjected to FACS analysis. FACS plots (A) showing the expression of Ly6A and DPP4 on PDGFRA^+^ dFBs (gating strategy is shown in Figure S1A). (B) Stacked bar graphs showing the quantified percentages of various dFB subpopulations, including DPP4^+^Ly6A^med^ dFBs, DPP4^lo^Ly6A^+^ pAds and DPP4^+^Ly6A^hi^ APs in PDGFRA^+^ dFBs across the wound centre, edge and distal regions as indicated (*n* = 3/group). All error bars indicate mean ± SEM. (C, D) FACS plots (C) showing the expression of Ly6A and THY1 on PDGFRA^+^ dFBs (w.d. 26). (D) Bar graphs showing the quantified percentages of THY1^hi^Ly6A^hi^ APs in PDGFRA^+^ dFBs (*n* = 3/group). All error bars indicate mean ± SEM. ****p < 0.0001. (E) Immunostaining of THY1 (red), Ly6A (green) and DPP4 (blue), and nuclei were stained by DAPI (white). Scale bar, 200 μm. (F) Immunostaining of CD31 (red), ACTA2 (green) and DAPI (blue for nuclei). Scale bar, 200 μm. (G, H) FACS plots (G) showing the expression of ACTA2 and PDGFRA on CD45^−^ skin cells. Red box marks the ACTA2^+^PDGFRA^−^ pericytes. Bar graphs (H) showing the quantified percentages of ACTA2^+^PDGFRA^−^ pericytes (*n* = 3/group). All error bars indicate mean ± SEM. *p < 0.05; **p < 0.01; ***p < 0.001.

Next, we aimed to characterise how dFBs, particularly adipocyte lineage cells, are dysregulated in the murine WISF model. PDGFRA^+^Ly6A^+^DPP4^+^ cells have been recently identified as hypodermal APs poised to differentiate into Ly6A^+^DPP4^−^ pAds and then into PDGFRA^−^ Ly6A^−^ FABP4^+^ adipocytes.^14^, ^35^, ^36^ Flow cytometry (fluorescence‐activated cell sorting, FACS) analysis of the PDGFRA^+^CD45^−^ dFBs (Figure S1A and Figure 2A,B) showed that the fibrotic centre scar was enriched with DPP4^+^Ly6A^hi^ dFBs, representing uncommitted HI‐AP, whereas the edge tissue was enriched with DPP4^lo^Ly6A^+^ dFBs, representing committed pAds.^14^ Furthermore, thymus cell antigen 1 (THY1) expression, an adipogenic inhibitor expressed on APs and pAds,^37^, ^38^, ^39^, ^40^ was markedly increased in the scar tissue compared to the control skin tissue (Figure 2C,D). Immunostaining revealed that in the distal control homeostatic skin, Ly6A^+^DPP4^+^ HI‐APs were confined to the hypodermal fascia layer, and Ly6A^+^DPP4^−^ pAds were exclusively found in the lower dermal dWAT layer (Figure 2E). In contrast, in the central scar tissue, an expansion of the hypodermal Ly6A^+^DPP4^+^ HI‐AP layer was observed, and THY1 expression notably increased in both HI‐APs and pAds (Figure 2E).

Hyperplasia of pericytes and perivascular smooth muscle‐like mural cells in the upper skin dermis is a characteristic feature of scleroderma or keloids.^41^ We found that ACTA2^+^ and CD31^+^ microvascular structures were abundantly detected in the scar centre, whereas the ACTA2^+^ structures in the control skin were mostly CD31^−^ arrector pili muscle structures (Figure 2F; immunostaining). Flow cytometry analysis further confirmed that the number of ACTA2^+^PDGFRA^−^ cells expressing THY1 but not Ly6A, representing perivascular pericytes,^42^, ^43^ significantly increased in the centre scar tissue compared to the edge or distal control skin (Figure 2G,H; Figure S1B).

Overall, we showed that wound‐induced skin fibrosis was associated with the loss of dermal adipocytes and abnormal accumulation of hypodermal APs, suggesting that blocking the adipogenic potential of interstitial adipogenic fibroblasts may help drive fibrotic skin pathology.

### 3.3 Reduced accumulation of HI‐APs and micro‐vessels in WIHN compared to WISF

**FIGURE 3:**
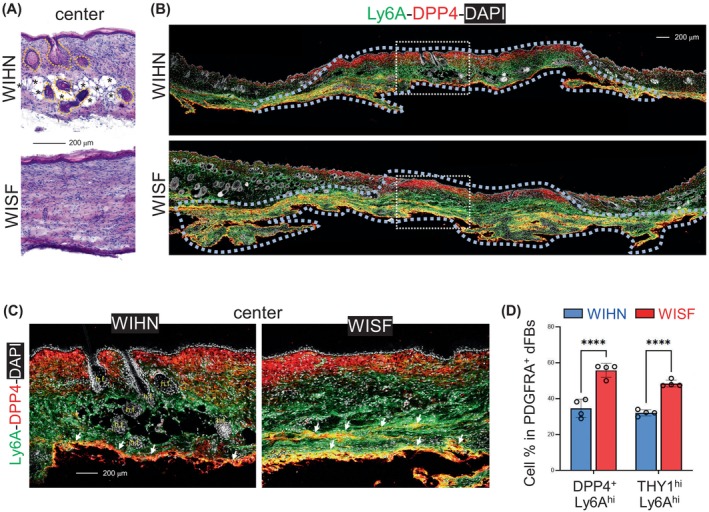
Reduced accumulation of HI‐APs and micro‐vessels in WIHN compared to WISF. Mice at 3 weeks (for WIHN) or 8 weeks (for WISF) of age were subjected to large skin wounding models as described in Figure 1, and wound samples were collected for analysis at w.d. 26. (A). HE staining of the WIHN or WISF centre scar tissues as indicated. Zoom‐in panels were shown on the right. Hair follicles were circled by dotted lines, and adipocytes are indicated by asterisks. Scale bar, 200 μm. (B, C). Immunostaining of DPP4 (red), Ly6A (green) and DAPI (white). Scale bar, 200 μm. Wound centre scar regions were marked by dotted‐lines. Zoom‐in panels were shown in C. Hair follicles were marked as ‘h.f.’, and DPP4^+^Ly6A^+^ HI‐APs were marked by white arrows. Scale bar, 200 μm. (D). Quantified bar graphs (see FACS plots in Figure S2B,C) showing the percentage of DPP4^+^Ly6A^hi^ or THY1^hi^Ly6A^hi^ APs in PDGFRA^+^ dFBs in the wound centre of WIHN or WISF tissues as indicated (*n* = 4/group). All error bars indicate mean ± SEM. ****p < 0.0001.

To elucidate the differences in HI‐AP accumulation between the WIHN and WISF models, large excisional wounds were performed in mice at 3 weeks (for WIHN) and 2 months (for WISF). HE staining confirmed that hair follicles and intradermal adipocytes were present in the wound centre regions of WIHN but not WISF samples (Figure 3A; Figure S2A). Immunostaining revealed that the centre of WISF was enriched with an expanded hypodermal layer of Ly6A^+^DPP4^+^ HI‐APs, whereas in the WIHN model, these cells were confined to a thin hypodermal layer (Figure 3B,C). Furthermore, FACS analysis showed that the percentages of DPP4^+^Ly6A^hi^ or THY1^hi^Ly6A^hi^ cells were significantly higher in the WISF compared to the WIHN scar tissues (Figure S2B,C; Figure 3D).

We have shown that WISF is enriched with CD31^+^ACTA2^+^ micro‐vessels (Figure 2). Here, immunostaining analysis revealed a shift in the ACTA2 signal from predominantly being present on CD31^+^ micro‐vessels in the WISF to being primarily associated with PDGFRA^+^CD31^−^ dermal sheath fibroblasts surrounding hair follicles in the WIHN (Figure S2D). Furthermore, numerous CD31^+^ACTA2^−^ micro‐vessels were detected in the WIHN scars (Figure S2D). Together, these results suggest that the wound‐induced abnormal accumulation of HI‐APs and CD31^+^ACTA2^+^ micro‐vessels are alleviated in younger mice, leading to more efficient regeneration of hair and adipocytes during wound regeneration.

### 3.4 Dermal adipocytes are absent in fibrotic human skin diseases

To determine how the mouse fibrotic phenotype is relevant to human fibrotic skin diseases, we analysed skin sections from keloids and scleroderma, two fibrotic skin disorders characterised by excessive ECM deposition and fewer skin appendages.^32^, ^33^, ^44^ Keloids are benign fibroproliferative tumours that result from an abnormal wound‐healing response in dermal injury.^3^ Scleroderma is a non‐tumorous skin lesion characterised by inflammation and fibrosis of the skin and subcutaneous adipose tissue.^45^

**FIGURE 4:**
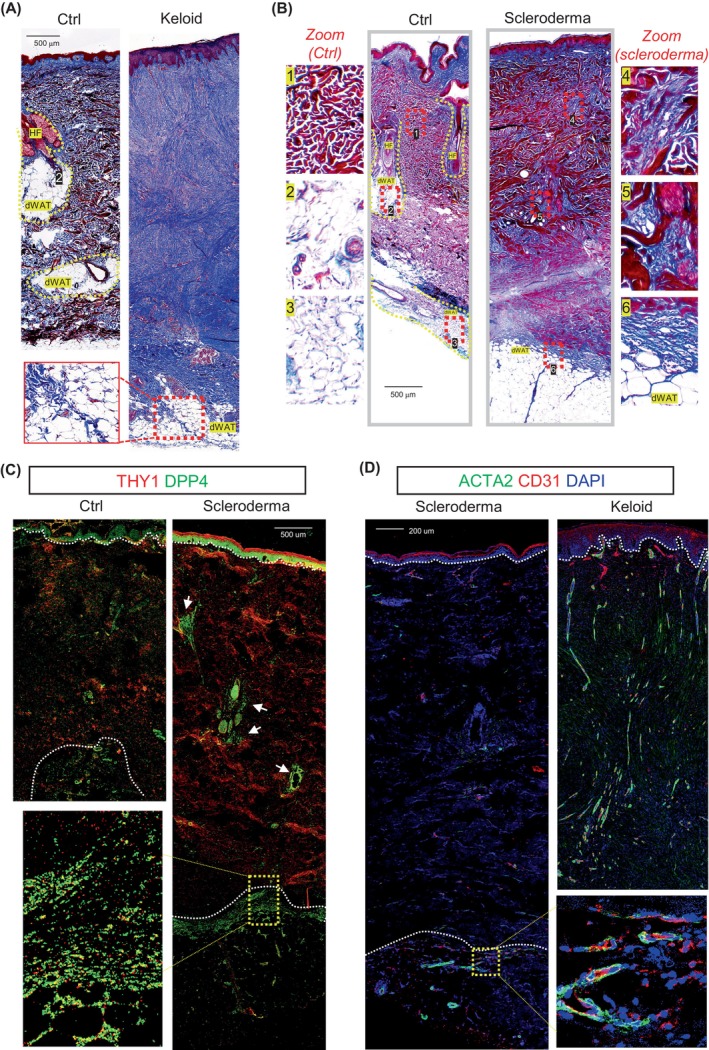
Dermal adipocytes are absent in the fibrotic human skin diseases. (A). Masson (collagen in blue) staining of healthy control and keloid skin sections as indicated. Three zoom‐in areas were shown in the lower left panel marked by dotted‐red box. Scale bar, 500 μm. (B). Masson (collagen in blue) staining of healthy control and scleroderma skin sections as indicated. zoom‐in areas of the control or scleroderma were shown on the left or right panel as indicated. Scale bar, 500 μm. (C) Immunostaining of DPP4 (green) and THY1 (red) of healthy control and scleroderma biopsies. White dashed lines marks the interface between the epidermis and dermis or the dermis and WAT tissue. Scale bar, 500 μm. (D) Immunostaining of DPP4 (green) and THY1 (red) in Scleroderma or keloid skin sections as indicated. Scale bar, 200 μm.

We found that adipocytes infiltrated the skin dermis (i.e., dermal adipocytes) in the healthy control skin samples, but they were largely lost in the dermis of keloids or scleroderma (Figure 4A,B). Furthermore, dermal adipocyte loss was accompanied by heavy deposition of collagen bundles within the dermal interstitial space (Figure 4A,B). In both keloid and scleroderma, long and linearised collagen bundles were detected at the interface between the dermis and hypodermal adipose tissue (Figure 4A,B).

Here, immunostaining of human scleroderma skin sections showed that DPP4^+^THY1^+^ fibroblasts accumulated in the dermal interstitial spaces and at the interface between the dermis and hypodermal adipose tissue (Figure 4C). Furthermore, CD31 + ACTA2+ microvascular structures were abundantly detected in keloid skin dermis and in the hypodermal region of the scleroderma skin sections (Figure 4D). These human results are highly consistent with the results from the WISF model.

### 3.5 The differentiation trajectory of HI‐APs during wound regeneration

**FIGURE 5:**
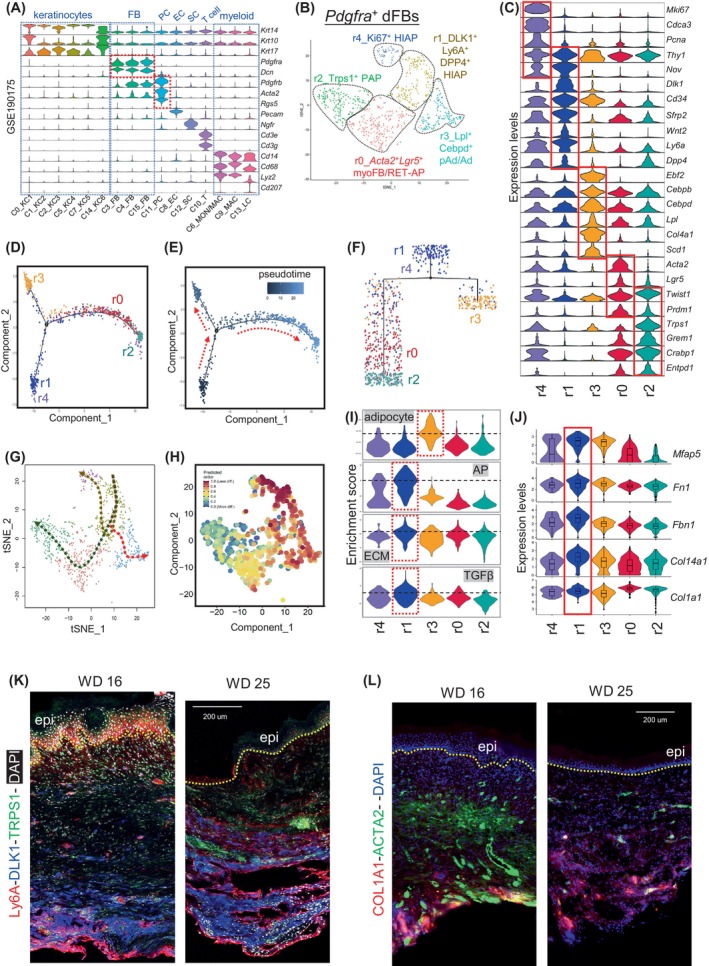
The differentiation trajectory of HI‐APs during wound regeneration. (A) Violin plots showing the expression of marker genes for each cell clusters of total cells from wound centre scar tissue (GSE190175). FB, fibroblasts; PC, pericytes; EC, endothelial cells; SC, Schwann cells. (B) tSNE plot showing the distribution of the five *Pdgfra* ^+^ dFB sub‐clusters. (C) Violin plots showing the expression of differentially expressed genes for each *Pdgfra* ^+^ dFB sub‐clusters. (D–F) Monocle 2‐based trajectory analysis annotated by cell clusters (D), pseudotime analysis (E), and hierarchical clustering annotated by cell clusters (F) of *Pdgfra* ^+^ dFB sub‐clusters. (G) Slingshot‐based lineage and pseudotime analysis predicting the three cell lineages and differentiation trajectory of *Pdgfra* ^+^ dFB sub‐clusters. (H) CytoTRACE analysis predicting the differentiation state of each *Pdgfra* ^+^ dFB sub‐clusters. diff, differentiated. (I) Violin plots showing the enrichment scores of gene‐sets related to adipocytes, adipocyte progentiors (AP), extracellular matrix (ECM), and TGFβ pathway in each dFB cluster. (J) Violin plots showing the expression of indicated ECM genes in each dFB cluster. (K, L) Immunostaining of the WISF centre scar tissues (w.d. 16 or w.d. 25) showing the expression of Ly6A (red), DLK1 (blue), TRPS1 (green) and DAPI (white) in K, and COL1A1 (red), ACTA2 (green) and DAPI (blue) in L. Scale bars, 200 μm.

To further determine the heterogeneity and differentiation trajectory of fibroblasts in healing wounds, we re‐analysed single‐cell RNA‐seq (Sc‐RNAseq) data from wound day (w.d.) 14 scar tissue (GSE190175),^46^ representing an actively healing early wound scar. A gene expression analysis of relevant markers in all cells identified *Krt14/Krt10* ^+^ keratinocytes, *Pdgfra/Dcn* ^+^ fibroblasts, *Rgs5* ^+^ pericytes, *Pecam1* ^+^ endothelial cells, *Ngfr* ^+^ Schwann cells, *Cd14/Cd68* ^+^ myeloid immune cells and *Cd3* ^+^ T cells (Figure S3A; Figure 5A). Similar to the FACS and staining results shown in Figure 2, violin plots of the Sc‐RNAseq data confirmed that the pericyte cluster cells were *Pdgfra* ^*−*^ *Pdgfrb* ^+^ *Acta2* ^+^ (Figure 5A).

Re‐clustering of the *Pdgfra* ^+^ fibroblasts yielded five sub‐clusters based on our recently established dFB marker panels (Figure 5B,C; Figure S3B), including the r1_HI‐APs (*Dlk* ^+^ *Ly6a* ^+^ *Dpp4* ^+^), r4_proliferative HI‐APs (*Ki67* ^+^ *Ly6a* ^+^ *Dpp4* ^+^), r3_pAd/Ad (*Lpl* ^+^ *Cebpd* ^+^), r0_*Acta2* ^+^ *Lgr5* ^+^ (myofibroblasts/myoFB; reticular/RET‐AP) and r2_papillary/PAP fibroblasts (*Trps1* ^+^ *Grem1* ^+^).^14^ Notably, r1_HI‐AP cells co‐express a panel of genes related to stem cells and APs, including *Nov*, *Cd34*, *Wnt2* and *Sfrp2*,^14^, ^47^, ^48^, ^49^, ^50^, ^51^ whereas r3_pAd/Ad cells co‐express genes related to adipogenesis, including *Ebf2, Col4a1*, and *Scd1*.^13^, ^52^, ^53^ A Monocle 2 trajectory analysis grouped these dFB clusters into three distinct developmental cell states: r1 and r4 in state 1, r3 in state 2 and r0 and r2 in state 3 (Figure 5D). Furthermore, several developmental trajectory analyses, including Monocle2 pseudotime (Figure 5E), tree plot (Figure 5F), slingshot‐based lineage/pseudotime^23^ (Figure 5G), and CytoTRACE^24^ (Figure 5H) analyses, all predicted that the r1_HI‐APs were the origin of the differentiation trajectory, differentiating and branching into state 2 (r3) and state 3 (r0 and r2) cells. Gene set enrichment analysis revealed that while adipocyte‐related genes were enriched in the r3 cluster, the r1_HI‐AP cluster was enriched with genes related to the ECM and transforming growth factor beta (TGFβ) (Figure 5I) and highly expressed a panel of ECM genes, including *Mfap5, Fn1, Fbn1, Col14a1* and *Col1a1* (Figure 5J). Immunostaining verified that early wound scar tissue (w.d. 16) was layered with Ly6A^+^DLK1^+^ HI‐APs in the hypodermis, ACTA2^+^ in the mid‐dermis, and TRPS1^+^ fibroblasts in the upper papillary dermis, whereas the static w.d. 25 scar tissue was enriched with Ly6A^+^DLK1^+^ HI‐APs that expressed type‐1 collagen (COL1A1) from the hypodermis to mid‐dermis (Figure 5K,L). These results suggest that the differentiation trajectory of HI‐APs may be blocked in the regenerating wound scar, resulting in the accumulation of ECM‐producing HI‐APs and the development of a fibrotic skin pathology.

### 3.6 TGFβ pathway is the key signal for maintaining fibrogenic function and inhibiting adipogenic function of dermal APs

**FIGURE 6:**
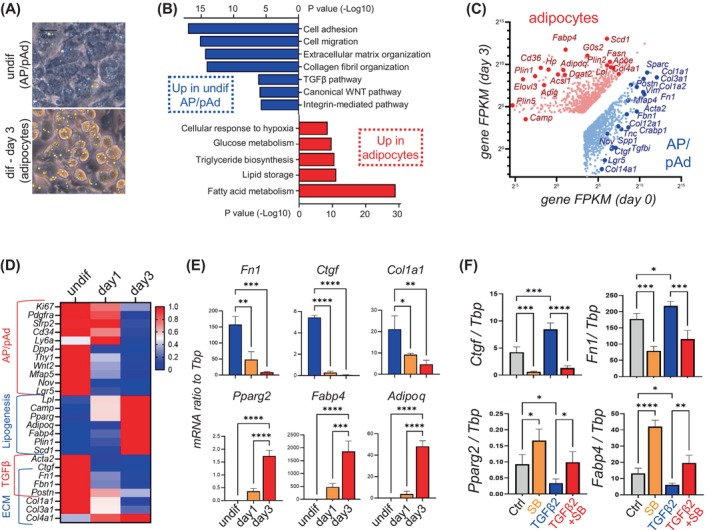
TGFβ pathway is the key signal for maintaining fibrogenic function and inhibiting adipogenic function of dermal APs. (A–E) Primary dermal AP/pAds were treated with adipocyte differentiation cocktail for 3 days before cells were collected for analysis. (A) Phase contrast images showing formation of lipid‐laden adipocytes at day 3 post‐differentiation. (B) GO pathway analysis showing top downregulated (blue) or upregulated (red) pathways in differentiated adipocytes compared to undifferentiated AP/pAds. (C) Scatter plot showing top differentially expressed genes in adipocytes and AP/pAds. (D, E) Heatmap (D) or qRT‐PCR (E) showing the mRNA expression kinetics of listed genes during adipocyte differentiation time course (*n* = 3/group). All error bars indicate mean ± SEM. *p < 0.05; **p < 0.01; ***p < 0.001; ****p < 0.0001. (F) qRT‐PCR analysis of key fibrotic and adipocyte‐related genes in neonatal AP/pAds treated with TGFβ ± TGFBR inhibitor (SB431542, SB) (n = 3/group). All error bars indicate mean ± SEM. *p < 0.05; **p < 0.01; ***p < 0.001; ****p < 0.0001.

Next, to investigate how adipogenic differentiation of AP/pAds influences ECM deposition and fibrogenesis, primary dermal AP/pAds were treated with an adipocyte differentiation cocktail for 3 days to induce differentiation into lipid‐laden adipocytes (Figure 6A), as previously described.^19^, ^39^ The Gene Ontology (GO) pathway analysis identified cell adhesion, migration, ECM organisation, and the TGFβ pathway among the top upregulated pathways, and fatty acid metabolism, lipid storage, triglyceride and glucose metabolism, and cellular response to hypoxia as the top downregulated pathways in the undifferentiated AP/pAds compared to differentiated adipocytes (Figure 6B).

In addition, scatter plots of differentially expressed genes (DEGs) showed that several ECM‐related genes (*Col1a1*, *Col3a1*, *Col14a1 and Fn1*) and TGFβ‐related genes (*Acta2*, *Ctgf*, *Postn*, *Tgfbi* and *Spp1*) were among the top DEGs in undifferentiated AP/pAds. In contrast, several adipocyte‐marker genes (*Fabp4*, *Adipoq*, *Plin* and *Camp*) and lipid‐metabolism genes (*Scd1*, *Dgat*, *Lpl*, *Cd36* and *Elovl3*) were among the top DEGs in differentiated adipocytes (Figure 6C). qRT‐PCR confirmed that the time‐dependent decrease in the expression of AP/pAd marker genes and ECM/TGFβ genes was accompanied by increased expression of adipocyte/lipogenesis genes during the in vitro differentiation of AP/pAds to adipocytes (Figure 6D,E; Figure S4). These results indicate that reinforcing the adipogenic differentiation programme in APs/pAds dampens the fibrogenic function of these progenitors by inhibiting the TGFβ pathway.

TGFβ is the central cytokine that drives fibrosis in scleroderma and keloids.^54^, ^55^ We previously identified TGFβ as the key negative regulator of dermal adipogenesis during ageing or diet‐induced obesity.^19^, ^39^ Here, we demonstrated that treating AP/pAds with TGFβ2 increased the expression levels of fibrosis/ECM‐related genes but suppressed adipocyte‐related genes, which could be largely reversed by adding a specific pharmacological inhibitor of TGFβ receptor (TGFBR), SB431542^39^, ^56^ (Figure 6F). Furthermore, TGFBR inhibitor (TGFBRi) treatment alone robustly suppressed basal levels of ECM/fibrosis‐related genes and increased adipocyte genes (Figure 6F), demonstrating that TGFβ plays a central role in maintaining the fibrogenic potential of APs by preventing these cells from differentiating into adipocytes.

### 3.7 TGFβ pathway is the key inhibitory signal for dermal adipogenesis in the WISF model

**FIGURE 7:**
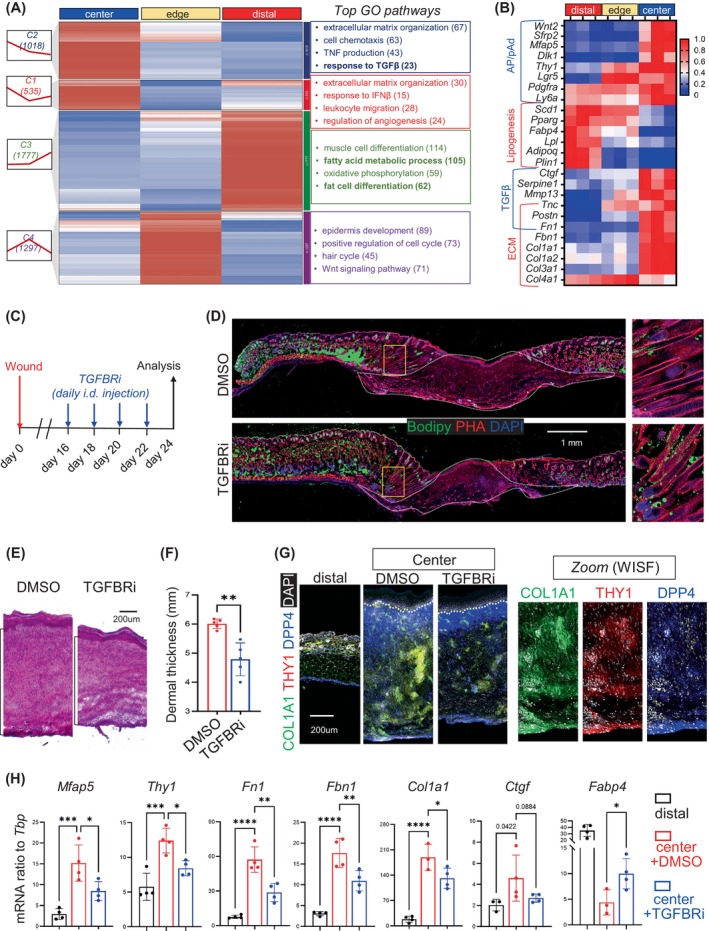
TGFβ pathway is the key inhibitory signal for dermal adipogenesis in the WISF model. (A) ClusterGVis analyses of the wound centre, edge and distal skin RNAseq data identified four gene clusters with distinct expression dynamics. Left panel: the number and expression pattern of genes in each cluster. Right panel: GO analyses of the relevant biological processes for each cluster. (B) Heatmap showing the relative expression of indicated genes. (C–H) Mature adult mice were administrated i.d. with TGFBR inhibitor (SB) or DMSO during the course of WISF, and wounds were collected at w.d. 24. (D) Bodipy staining of skin wounds. Zoom‐in images cropped from the edge area are shown on the right. Scale bar, 1 mm. (E, F) HE staining of the centre scar tissue (E), and quantified results showing the thickness of skin dermis is shown in F (*n* = 5/group). Scale bar, 200 μm. (G) Immunostaining of COL1A1 (green), THY1 (red), DPP4 (blue) and DAPI (white). Scale bar, 200 μm. (H) qRT‐PCR analysis of key fibrotic or adipocyte‐related genes as indicated (*n* = 3 ~ 4/group). All error bars indicate mean ± SEM. *p < 0.05; **p < 0.01; ***p < 0.001; ****p < 0.0001.

Next, we investigated the role of TGFβ in maintaining AP and suppressing adipogenesis in the WISF model. First, a GO pathway analysis of the DEGs between distal, edge and centre skin wound regions showed that the centre scar was enriched with genes related to ECM organisation, response to TGFβ, regulation of angiogenesis and several immune pathways (Figure 7A). In contrast, the distal region was enriched with genes related to fatty acid metabolic processes, oxidative phosphorylation and fat cell differentiation, whereas the edge region was enriched with genes related to epidermal development, cell cycle, hair cycle and WNT signalling (Figure 7A). A gene expression heatmap and qRT‐PCR results illustrated that while lipogenesis/adipocyte genes were suppressed in the wound centre, genes related to AP/pAd, ECM and TGFβ pathway were upregulated in the centre compared to the distal control and edge samples (Figure 7B; Figure S5). These results indicate that TGFβ signalling is highly active in the scar tissue.

To investigate whether targeted inhibition of TGFBR can alleviate skin fibrosis, we intradermally injected TGFBRi (SB431542) into the wound from w.d. 16–22 (Figure 7C). TGFBRi treatment did not robustly enhance lipogenesis in the wound scar, but it enhanced lipogenesis at the wound edge (Figure 7D; BODIPY stain). In the wound centre, TGFBRi treatment significantly reduced the scar thickness and cellular density of the hypodermis (Figure 7E,F). In addition, TGFBRi suppressed COL1A1 expression in DPP4^+^THY1^+^ APs (Figure 7G) in the lower dermis of the scar tissue. Consequently, HI‐AP marker (*Mfap5* and *Thy1*), ECM (*Fn1*, *Fbn1* and *Col1a1*), and TGFβ target gene (*Ctgf*) expression levels were suppressed, whereas adipocyte gene expression (*Fabp4*) was enhanced in the wound centre tissues treated with TGFBRi compared to a DMSO control (Figure 7H). These results demonstrate that the TGFβ pathway is the key inhibitory signal for dermal adipogenesis in the WISF model.

### 3.8 Gene expression analysis of AP‐, ECM‐ and TGFβ pathway‐related genes in human scleroderma

**FIGURE 8:**
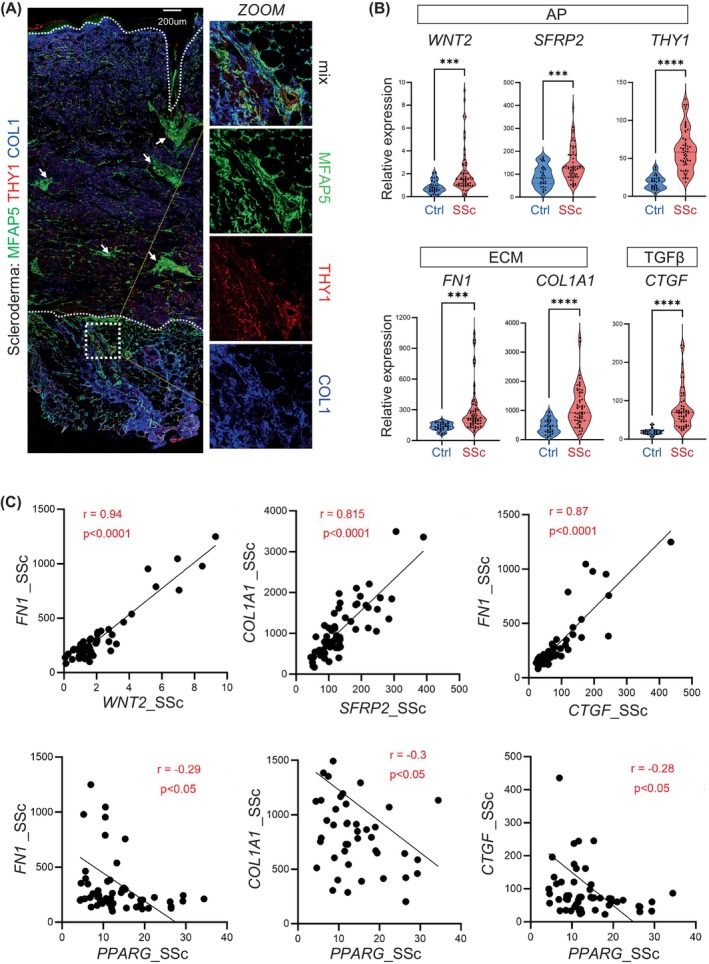
Gene expression analysis of AP‐, ECM‐ and TGFβ pathway‐related genes in human scleroderma. (A) Immunostaining of MFAP5 (green), THY1 (red) and COL1 (blue) of scleroderma skin sections. White dashed lines marks the interface between the epidermis and dermis or the dermis and WAT tissue. Zoom‐in images with mixed‐ or single‐colour channels are shown on the right panel. Scale bar, 200 μm. (B) Violin plots showing the expression levels (Fragments Per Kilobase of transcript per Million mapped reads/FPKM values) of genes related to adipocyte progenitors/AP, extracellular matrix/ECM, and TGFβ pathway as shown in healthy controls (*n* = 33) or scleroderma (SSc) (*n* = 58) skin samples. All error bars indicate mean ± SEM. ***p < 0.001; ****p < 0.0001. (C) Correlation expression plots of indicated genes. Linear correlation analysis was performed by Pearson correlation coefficient method. The r value represents the correlation coefficient strength, and *p* value assesses the statistic significance of the correlation.

Immunostaining of the human scleroderma skin sections revealed that ECM protein MFAP5 were enriched in the interstitial spaces (white arrows) and the hypodermal interphase space between the dermis and dermal adipose tissue, where THY1 and COL1 co‐expressed (Figure 8A). These results indicate that the interstitial and hypodermal dermal fibroblasts are key cellular source of MFAP5 and COL1 in human fibrotic skin.

Next, we analysed data from the skin biopsy RNA‐seq database (GSE130955^20^) to identify early factors driving disease pathogenesis. This database contained 33 healthy controls and 58 patients in the Prospective Registry of Early Systemic Sclerosis (i.e., PRESS) cohort, which only included patients in the early stages of diffuse cutaneous SSc (mean disease duration: 1.3 years). We found that AP (*WNT2, SFRP2* and *THY1*), ECM (*FN1*, *COL1A1* and *POSTN*) and TGFβ pathway (*CTGF/CCN2* and *SERPINE1*) gene expression levels were significantly higher in the SSc samples than in the healthy control samples (Figure 8B; Figure S6A). Furthermore, *FN1* and/or *COL1A1* expression positively correlated with *WNT2*, *SFRP2, THY1* and/or *CTGF* expression (Figure 8B; Figure S6B). In contrast, *FN1*, *COL1A1* and *CTGF* expression negatively correlated with *PPARG* expression (Figure 8C). These results suggest that sustained TGFβ pathway activation and adipogenic PPARγ pathway inhibition may impair the differentiation function of dermal APs, leading to uncontrolled dermal accumulation of ECM‐producing APs. These observations in human SSc are highly consistent with our WISF mouse model results.

## 4 DISCUSSION AND CONCLUSION

In this study, we established an adult WISF mouse model in which the central scar tissue was characterised by excessive ECM deposition and a lack of skin appendages, such as hair follicles and adipocytes, which are also characteristics of human fibrotic skin diseases, such as scleroderma and keloids. FACS, staining, and single‐cell RNA‐seq analyses showed that the central scar tissue was enriched with pathological microvascular structures and ECM‐producing APs in the hypodermis. The TGFβ pathway was identified as the key pathway that blocks the differentiation of hypodermal APs into adipocytes, resulting in excessive ECM accumulation and the development of the fibrotic pathology of the WISF model. In contrast to the extensively used intradermal bleomycin injection mouse model for skin fibrosis,^57^ this WISF model does not require daily injections and is, therefore, less labour‐intensive and more natural and physiologically relevant for skin fibrosis and the development of disease‐modifying therapies.

The procedure used for the WISF model was identical to that used for the WIHN model, except that we used mice aged 8 weeks instead of 3 weeks, which is typical for the WIHN model to observe efficient wound‐induced hair neogenesis.^26^, ^27^, ^28^, ^31^ This age‐dependent gain of wound‐induced fibrotic phenotype might be due to age‐related activation of the TGFβ pathway in dFBs. Activation of TGFβ signalling has also been linked with the development of several ageing‐associated disorders, such as tissue fibrosis, Alzheimer's disease, muscle atrophy, obesity and skin infection.^39^, ^58^ We have previously shown that dFBs isolated from neonatal and young mice are highly adipogenic, whereas dFBs (including APs) gradually lose the adipogenic potential and gain fibrotic phenotype during adulthood and ageing.^14^, ^39^ Furthermore, we showed that this age‐related adipogenic to fibrotic switch of dFBs was driven by age‐dependent activation of the TGFβ pathway.^14^ However, future study is still needed to investigate how dysregulation of the TGFβ pathway in APs contributes to the age‐dependent loss of wound‐induced hair neogenesis and the gain of wound‐induced fibrosis.

New studies have revealed that the hypodermal interstitium, a newly recognised skin structure enriched with progenitors, plays an essential role in skin wound healing and fibrosis.^14^, ^17^, ^18^ A subset of hypodermal fascia DPP4^+^Ly6A^+^ and CD201^+^ fibroblasts are known to have remarkable migratory and reparative potential during wound healing, and genetic ablation of fascia fibroblasts or blocking these cells from migrating to wound sites leads to chronic wounds.^17^, ^18^, ^59^, ^60^ SFRP2^+^ fascia fibroblasts have been identified as the TH2‐interacting fascial fibroblasts (TIFFs) that expand in response to TH2 cytokines to form hypodermal fibrous bands, contributing to the development of skin fibrosis under inflammatory condition in both mouse and human skin.^61^ Our recent study showed that sorted DPP4^+^Ly6A^+^ dFBs are both adipogenic and osteogenic, confirming that these are multipotent progenitors.^14^ Our study highlighted that the hypodermal DPP4^+^Ly6A^+^ APs are indispensable in WISF. Our results also indicated that TGFβ‐mediated blockage of the adipogenic function of these APs caused AP accumulation, uncontrolled ECM deposition, and the loss of dermal adipocytes in the WISF model.

Moreover, we identified HI‐AP as the primary dermal source of several pro‐fibrotic ECM molecules, including fibronectin (FN1), fibrillin‐1 (FBN1) and microfibril‐associated protein 5 (MFAP5), and that inhibiting the TGFBR pathway inhibits their expression. FN1 is the earliest and most abundant ECM component in the provisional matrix during wound healing. Furthermore, it connects to other ECM molecules, such as collagens and FBN1, and mediates cell adhesion and migration through the intergrin‐FN interaction, mechanochemical signal transduction, fibroblast proliferation and TGFβ responsiveness.^15^, ^62^, ^63^ Polymerisation of FBN1 forms the microfibril structure that controls the bioavailability of several growth factors, including TGFβ.^15^ An FBN1 mutation releases TGFβ, and FBN1 overexpression activates the TGFBR‐SMAD2/3 pathway via the integrin αvβ6 signalling.^64^ A recent report showed that MFAP5 is upregulated during the proliferative and remodelling phases of wound healing and that neutralising MFAP5 decreases collagen deposition and refines angiogenesis.^65^ In vitro, MFAP5 is induced by TGFβ, and it enhances fibroblast migration, pro‐fibrotic gene expression, and collagen contractility, indicating that MFAP5 might be a key player in scar formation during wound healing.^65^ These results suggest that reinforcing the differentiation of HI‐APs into adipocytes by inhibiting the TGFBR pathway may dampen the expression of these pro‐fibrotic ECM molecules, thus improving WISF.

In our study of the murine WISF model, we identified a distinct population of HI‐APs, characterised by the co‐expression of *Ly6a, Dpp4, Thy1, Sfrp2* and a panel of ECM genes (*Col1a1, Col14a1, Fn1* and *Mfap5*). Although the murine Ly6A does not have a human ortholog, we observed that in the context of systemic sclerosis (SSc), human hypodermal interstitial fibroblasts co‐expressed DPP4, THY1 and high levels of COL1 and MAFP5, resembling the murine HI‐APs. The staining pattern of the THY1^+^MFAP5^+^ fibroblasts we observed in SSc is similar to that of perivascular adventitial fibroblasts, which are characterised by expression of THY1 (CD90), VCAM1 and MFAP5 in the fibrotic human skin dermis, as reported by Barron et al.^66^ Consistent with our results, ScRNAseq analysis of normal human abdominal skin samples has identified a distinct dFB subcluster that expresses DPP4, THY1, MFAP5, SFRP2.^67^ Among all dFB subtypes, this dFB subtype has a strongest association with ECM assembly, wound healing, and angiogenesis.^68^ Furthermore, ScRNAseq analysis of SSc skin samples has identified a SFRP2^hi^/DPP4‐expressing progenitor subpopulation, with a potential role in driving SSc myofibroblast differentiation.^67^ Another human SSc study also identified a scar‐specific fibroblast subpopulation co‐expressing DPP4, THY1, SFRP2, COL1A1, COL14A1 and FN1.^69^ Functional assays further demonstrate that DPP4, the serine protease that is upregulated in both human and mouse scars, plays a pro‐fibrotic role by promoting TGFB‐mediated myfibroblast differentiation and over‐production of ECM components.^69^, ^70^ Collectively, these evidences suggest that the human profibrotic cells express a similar set of markers to those found in the pro‐fibrotic murine HI‐APs identified in our study.

This study has a few limitations. First, it lacks in vivo models for lineage tracing or ablation of HI‐APs to validate their function as the origin of dermal fibrosis and adipogenesis in WISF, although sorted Ly6A^+^DPP4^+^ HI‐APs are highly adipogenic in vitro.^14^ WNT2 is a highly specific marker for HI‐APs^14^; therefore *Wnt2‐Cre* mice can be generated to specific ablate or lineage trace HI‐APs in WISF in future study. Second, we showed that abnormal accumulation of pericytes is a key characteristics of the WISF model, but we did not further explore the role of pericytes in driving the fibrotic phenotype. Pericytes are multipotent cells with fibrogenic and adipogenic potential,^71^, ^72^, ^73^ and hyperplasia of dermal pericytes plays an indispensable role in neo‐vascularisation, synthesising ECM, regulating immune cell trafficking and activation, and secreting fibroblast‐activating cytokines and growth factors in wound healing, scleroderma and keloids.^43^, ^74^, ^75^ Future study is needed to identify key factor that inhibits the adipogenic potential and maintains the fibrogenic potential of pericytes in WISF.

In summary, this study established and characterised a WISF model in adult mice and highlighted how dysregulation of the adipogenesis programme in hypodermal APs plays a critical role in the excessive accumulation of ECM molecules during wound regeneration. Our study emphasises the importance of using mature adult mice to model WISF, which differentiates our approach from those focusing on hair neogenesis in younger mice. This WISF model will help researchers investigate how microvascular damage, pericytes, and hypodermal APs drive the fibrotic pathology during wound healing and study the mechanisms underlying age‐related dysregulation of wound‐induced hair neogenesis.

## AUTHOR CONTRIBUTIONS

*Methodology*: M.Y., L.S., S.W., Y.Y., L‐J. Z. *Investigation*: M.Y., L.S., S.W., J.M., X.J., W.Z., Y.Y., Xiaowei.Z., Z.T., Xinyuan. Z., W.L. *Conceptualisation*: M.Y., L.S., L‐J. Z. *Resources*: H‐J. W., S.J.; C.J. *Writing—original draft*: M.Y., L.S., S.W., L‐J. Z. *Writing—review & editing*: L‐J. Z.; *Supervision*: L‐J. Z.

## FUNDING INFORMATION

This study was supported by National Key R&D Program of China (2023YFC2508102 to L‐J.Z.), National Natural Science Foundation of China (82373879 and 81971551 to L‐J.Z. and 82103702 to S.L.), and the postdoctoral science foundation of China (2020M682095 to S.L.).

## CONFLICT OF INTEREST STATEMENT

The authors declare no conflict of interest.

## Supporting information


**TABLE S1.** List of abbreviations.
**TABLE S2.** List of protein and gene symbols.
**TABLE S3:** Antibodies used in this study.
**TABLE S4:** Specific primer sequences used in this study.
**FIGURE S1.** FACS analysis of dermal fibroblasts isolated from mouse skin wounds.
**FIGURE S2.** Reduced accumulation of HI‐APs and micro‐vessels in WIHN compared to WISF.
**FIGURE S3.** Single‐cell RNAseq of the wound‐induced centre scar skin tissue.
**FIGURE S4.** Analysis of gene expression during the in vitro adipogenic differentiation of dermal adipocyte progenitors.
**FIGURE S5.** Activation of fibrogenesis and inhibition of dermal adipogenesis in the WISF model.
**FIGURE S6.** Analysis of the expression of genes related to AP and ECM in human scleroderma.

## Data Availability

The data that support the findings of this study are available in single‐cell RNA‐seq dataset of the centre wound skin tissue at https://www.ncbi.nlm.nih.gov/geo/, reference number GSE190175. These data were derived from the following resources available in the public domain: GEO, https://www.ncbi.nlm.nih.gov/geo/query/acc.cgi?acc=GSE190175.
