# Supplementary material for: TGFβ‐mediated inhibition of hypodermal adipocyte progenitor differentiation promotes wound‐induced skin fibrosis
Source: Cell Prolif. 2024 Jul 29;58(1):e13722. doi: 10.1111/cpr.13722 (PMC11693572; doi:10.1111/cpr.13722)
Supplement: Supplementary file 1 — TABLE S1. List of abbreviations. TABLE S2. List of protein and gene symbols. TABLE S3: Antibodies used in this study. TABLE S4: Specific primer sequences used in this study. FIGURE S1. FACS analysis of dermal fibroblasts isolated from mouse skin wounds. FIGURE S2. Reduced accumulation of HI‐APs and micro‐vessels in WIHN compared to WISF. FIGURE S3. Single‐cell RNAseq of the wound‐induced centre scar skin tissue. FIGURE S4. Analysis of gene expression during the in vitro adipogenic differentiation of dermal adipocyte progenitors. FIGURE S5. Activation of fibrogenesis and inhibition of dermal adipogenesis in the WISF model. FIGURE S6. Analysis of the expression of genes related to AP and ECM in human scleroderma. [file CPR-58-e13722-s001.pdf]

Table S1. List of Abbreviations:

Abbreviations	Definitions
3W	3 weeks
8W	8 weeks
Ad	Adipocyte
ALP	Alkaline phosphatase
AP	Adipocyte progenitor
APM	Arrector pili muscle
DEG	Differentially expressed gene
dFB	Dermal fibroblast
diff	Differentiated
DMSO	Dimethyl sulfoxide
dWAT	Dermal white adipose tissue
EC	Endothelial cell
ECM	Extracellular matrix
Epi	Epidermis
FACS	Fluorescence activated cell sorting
FPKM	Fragments per kilobase of transcript per million mapped reads
GO	Gene ontology
H&E	Hematoxylin-eosin staining
HF	Hair follicle
HI	Hypodermal interstitium
HI-AP	Hypodermal interstitial adipocyte progenitor
i.d.	Intradermal injection
IHC	Immunohistochemistry
KC	Keratinocyte
LC	Langerhans cell
MAC	Macrophage
MON	Monocyte
myoFB	Myofibroblast
ORO	Oil-Red-O
pAd	Preadipocytes
PAP	Papillary
PC	Pericyte
PFA	Paraformaldehyde
PHA	Phalloidin
PRESS	Prospective registry of early systemic sclerosis
qRT-PCR	Quantitative reverse transcription - PCR
RET	Reticular
RNA-seq	RNA sequencing
SB	SB431542

Sc-RNAseq	Single cell RNA sequencing
SPF	Standard pathogen free
SSc	Scleroderma
TGFBR	TGFβ receptor
TGFBRi	TGFBR inhibitor
TIFF	TH2-interacting fascial fibroblast
t-SNE	t-distributed stochastic neighbor embedding
w.d.	Wound day
WIHN	Wound-induced hair follicle neogenesis
WISF	Wound-induced skin fibrosis

Table S2. List of protein and gene symbols:

**Proteins**	**Protein symbols**	**Gene symbols**
acyl-CoA synthetase long-chain family member 1	ACSL1	*Acsl1*
Actin Alpha 2	ACTA2	*Acta2*
adipogenin	ADIG	*Adig*
Adiponectin	ADIPOQ	*Adipoq*
apolipoprotein E	APOE	*Apoe*
Cathelicidin antimicrobial peptide	CAMP	*Camp*
CCN family member 2	CTGF	*Ccn2*
cellular communication network factor 3	CCN3/NOV	*Ccn3/Nov*
CD14 antigen	CD14	*Cd14*
CD207 Molecule	CD207	*Cd207*
T-cell surface glycoprotein CD3	CD3	*Cd3*
Hematopoietic progenitor cell antigen CD34	CD34	*Cd34*
Platelet glycoprotein 4	CD36	*Cd36*
CD68 antigen	CD68	*Cd68*
cell division cycle associated 3	CDCA3	*Cdca3*
CCAAT/enhancer-binding protein beta	CEBPB	*Cebpb*
CCAAT/enhancer binding protein delta	CEBPD	*Cebpd*
Collagen	COL	*Col*
Cellular retinoic acid-binding protein 1	CRABP1	*Crabp1*
decorin	DCN	*Dcn*
diacylglycerol O-acyltransferase	DGAT	*Dgat*
delta like non-canonical Notch ligand 1	DLK1	*Dlk1*
Dipeptidyl peptidase 4	DPP4	*Dpp4*
early B cell factor 2	EBF2	*Ebf2*
ELOVL fatty acid elongase 3	ELOVL3	*Elovl3*
Ectonucleoside triphosphate diphosphohydrolase 1	ENTPD1	*Entpd1*

fatty acid binding protein 4	FABP4	*Fabp4*
fatty acid synthase	FASN	*Fasn*
fibrillin 1	FBN1	*Fbn1*
fibronectin 1	FN1	*Fn1*
G0/G1 Switch 2	G0S2	*G0s2*
gremlin 1, DAN family BMP antagonist	GREM1	*Grem1*
keratin 14	KRT	*Krt*
Leucine-rich repeat-containing G-protein coupled receptor 5	LGR5	*Lgr5*
Lipoprotein lipase	LPL	*Lpl*
Lymphocyte antigen 6A-2/6E-1	Ly6A	*Ly6a*
Lysozyme C-2	LYZ2	*Lyz2*
microfibrillar associated protein 5	MFAP5	*Mfap5*
Proliferation marker protein Ki-67	Ki67	*Mki67*
matrix metallopeptidase 13	MMP13	*Mmp13*
nerve growth factor receptor	NGFR	*Ngfr*
Proliferating cell nuclear antigen	PCNA	*Pcna*
Platelet-derived growth factor receptor alpha	PDGFRA	*Pdgfra*
platelet derived growth factor receptor, beta polypeptide	PDGFRB	*Pdgfrb*
platelet/endothelial cell adhesion molecule 1	PECAM-1/CD31	*Pecam1*
Perilipin	PLIN	*Plin*
periostin, osteoblast specific factor	POSTN	*Postn*
Peroxisome proliferator-activated receptor gamma	PPARG	*Pparg*
PR/SET Domain 1	PRDM1	*Prdm1*
Receptor-type tyrosine-protein phosphatase C	CD45	*Ptprc*
regulator of G-protein signaling 5	RGS5	*Rgs5*
stearoyl-Coenzyme A desaturase 1	SCD1	*Scd1*
Serpin Family E Member 1	SERPINE1	*Serpine1*
Secreted Frizzled Related Protein 2	SFRP2	*Sfrp2*
secreted acidic cysteine rich glycoprotein	SPARC	*Sparc*
secreted phosphoprotein 1	SPP1	*Spp1*
TATA box binding protein	TBP	*Tbp*
Transforming growth factor beta	TGFB	*Tgfb*
Transforming growth factor-beta-induced protein igh3	TGFBI	*Tgfbi*
Thy-1 membrane glycoprotein	THY1	*Thy1*
tenascin C	TNC	*Tnc*
transcriptional repressor GATA binding 1	TRPS1	*Trps1*
twist basic helix-loop-helix transcription factor 1	TWIST1	*Twist1*
Vimentin	VIM	*Vim*
Wnt Family Member 2	WNT2	*Wnt2*

Table S3: Antibodies used in this study:

Antibodies	Vender	Catalog
PE anti-THY1	BioLegend	105308
BV605 anti-LY6A	BioLegend	108133
AF488 anti-SMA/ACTA2	eBioscience	53976082
Rabbit anti-COL1A1	abcam	b063-ab34710
APC anti-PDGFRA	eBioscience	17140181
PECy7 anti-CD45	Biolegend	147704
PerCP-Cy5.5 anti-DPP4	eBioscience	2142977
Rat anti-LY6A	R&D systems	MAB1226
Goat anti-DPP4	R&D systems	AF954-SP
Rat anti-CD31	ThermoFisher Scientific	14-0311-82
Anti-DLK1	R&D systems	AF8277
CD31 Polyclonal antibody	Proteintech	11265-1-AP
PE anti- human CD90 (Thy1)	BioLegend	328109
MFAP5 Polyclonal antibody	Proteintech	15727-1-AP
Goat Anti-Type Ⅰ Collagen	SouthernBiotech	1310-01
Alexa Fluor 647 AffiniPure Donkey Anti-Rat IgG (H+L)	Jackson ImmunoResearch	712-606-150
Alexa Fluor 647 AffiniPure Donkey Anti-Goat IgG (H+L)	Jackson ImmunoResearch	705-606-147
Alexa Fluor 488 AffiniPure Donkey Anti-Rat IgG (H+L)	Jackson ImmunoResearch	712-545-150
Alexa Fluor 488 AffiniPure Donkey Anti-Rabbit IgG (H+L)	Jackson ImmunoResearch	711-545-152
Cy™3 AffiniPure™ Donkey Anti-Rabbit IgG (H+L)	Jackson ImmunoResearch	711-165-152

Table S4: Specific primer sequences used in this study:

Gene	Strand	Primer sequence
Tbp	Forward	CCTTGTACCCTTCACCAATGAC
	Reverse	ACAGCCAAGATTCACGGTAGA
Col1a1	Forward	GCTCCTCTTAGGGGCCACT
	Reverse	ATTGGGGACCCTTAGGCCAT
Adipoq	Forward	CACACCAGGCCGTGATGGCA
	Reverse	GAAGCCCCGTGGCCCTTCAG
Fabp4	Forward	GTGGGAGTGGGCTTTGCCACA
	Reverse	CACCAGGGCCCCGCCATCTA
Pparg2	Forward	TCGCTGATGCACTGCCTATG
	Reverse	GAGAGGTCCACAGAGCTGATT
Ctgf	Forward	GGGCCTCTTCTGCGATTTC
	Reverse	ATCCAGGCAAGTGCATTGGTA
Fn1	Forward	TTCAAGTGTGATCCCCATGAAG
	Reverse	CAGGTCTACGGCAGTTGTCA
Mfap5	Forward	GTCTTGGCAATCAGCATCCC
	Reverse	CCAGATTAGGGTCGTCTGTGAAT
Fbn1	Forward	CATTCCTGTGGGGATGGATTC
	Reverse	TACGTGCAAGCACACCGATTT
Thy1	Forward	CCTTACCCTAGCCAACTTCAC
	Reverse	AGGATGTGTTCTGAACCAGC
Postn	Forward	TGGTATCAAGGTGCTATCTGCG
	Reverse	AATGCCCAGCGTGCCATAA

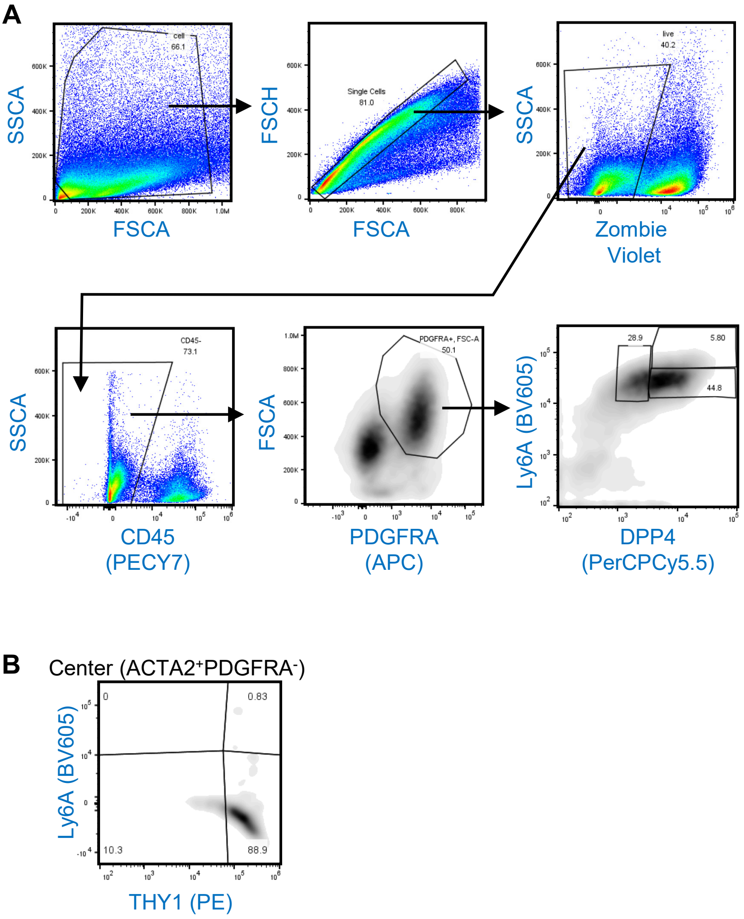

Figure S1. FACS analysis of dermal fibroblasts isolated from mouse skin wounds
(**A**) Gating strategy for FACS analysis of the surface expression of Ly6A and DPP4 on $CD45^-PDGFRA^+$ dermal fibroblasts shown in Figure 3A.
(**B**) FACS plot showing the surface expression of Ly6A and THY1 on $ACTA2^+PDGFRA^-$ pericytes in wound center tissue.

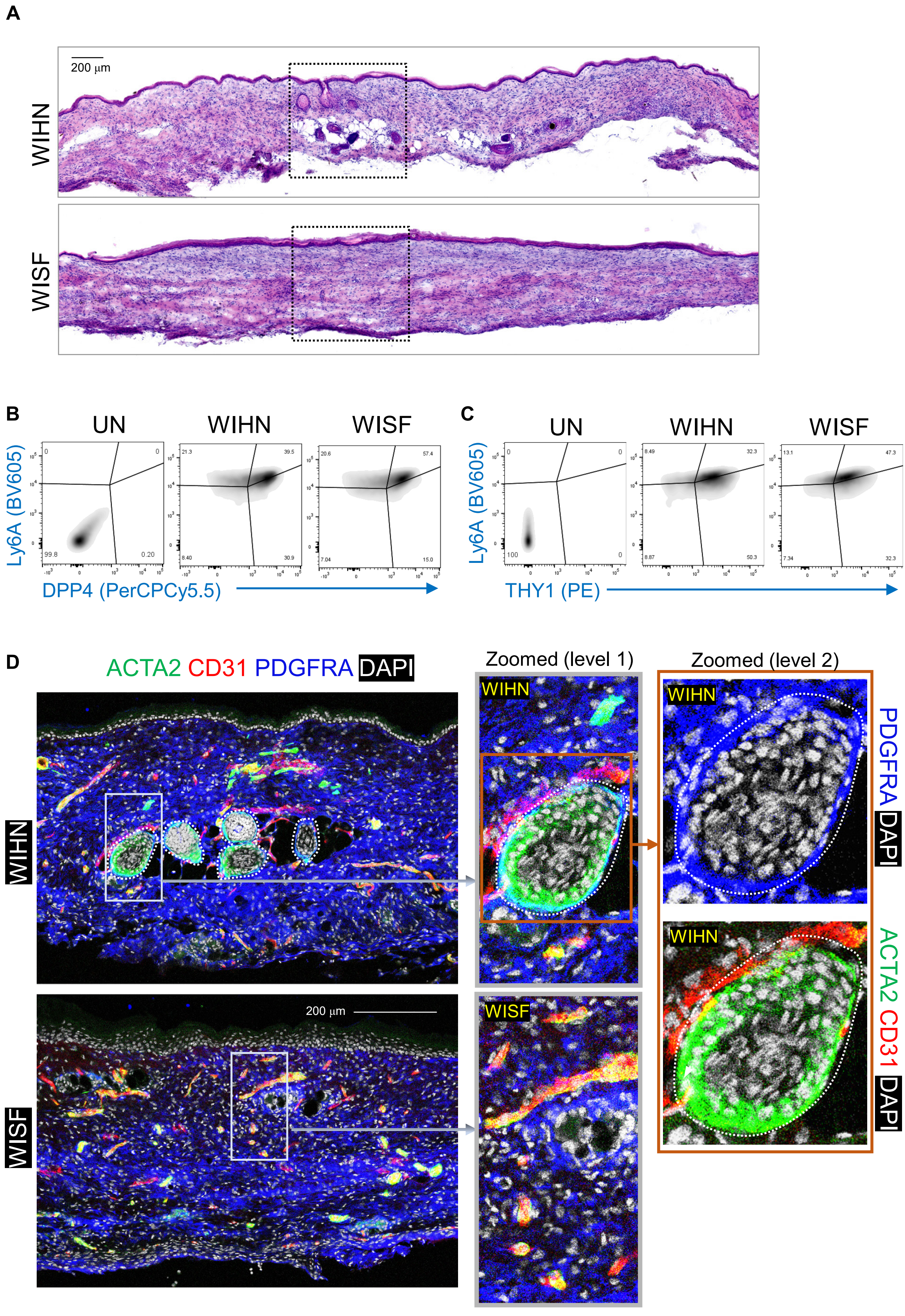
A
200 μm
WIHN
WISF
B
UN
WIHN
WISF
Ly6A (BV605)
DPP4 (PerCPCy5.5)
C
UN
WIHN
WISF
Ly6A (BV605)
THY1 (PE)
D
ACTA2 CD31 PDGFRA DAPI
Zoomed (level 1)
Zoomed (level 2)
WIHN
WISF
200 μm
PDGFRA DAPI
ACTA2 CD31 DAPI

Figure S2.

Figure S2. Reduced accumulation of HI-APs and micro-vessels in WIHN compared to WISF. Mice at 3 weeks (for WIHN) or 8 weeks (for WISF) of age were subjected to large skin wounding model, and wounds were collected at w.d. 26 for analysis.
(**A**) HE staining of the WIHN or WISF scar tissues as indicated. Zoom-in panels marked by dotted boxes were shown on Fig. 3A. Scale bar, 200 μm.
(**B-C**) Cells isolated from the center scar tissues were subjected to FACS analysis. FACS plot showing the surface expression of Ly6A and DPP4 (**B**), or Ly6A and THY1 (**C**) in PDGFRA$^+$CD45$^-$CD31$^-$ fibroblasts.
(**D**) Immunostaining of CD31 (red), ACTA2 (green), PDGFRA (blue), and DAPI (white for nuclei). Scale bar, 200 μm. Hair follicles were circled by dotted lines. Zoom-in panels were shown on the right as indicated.

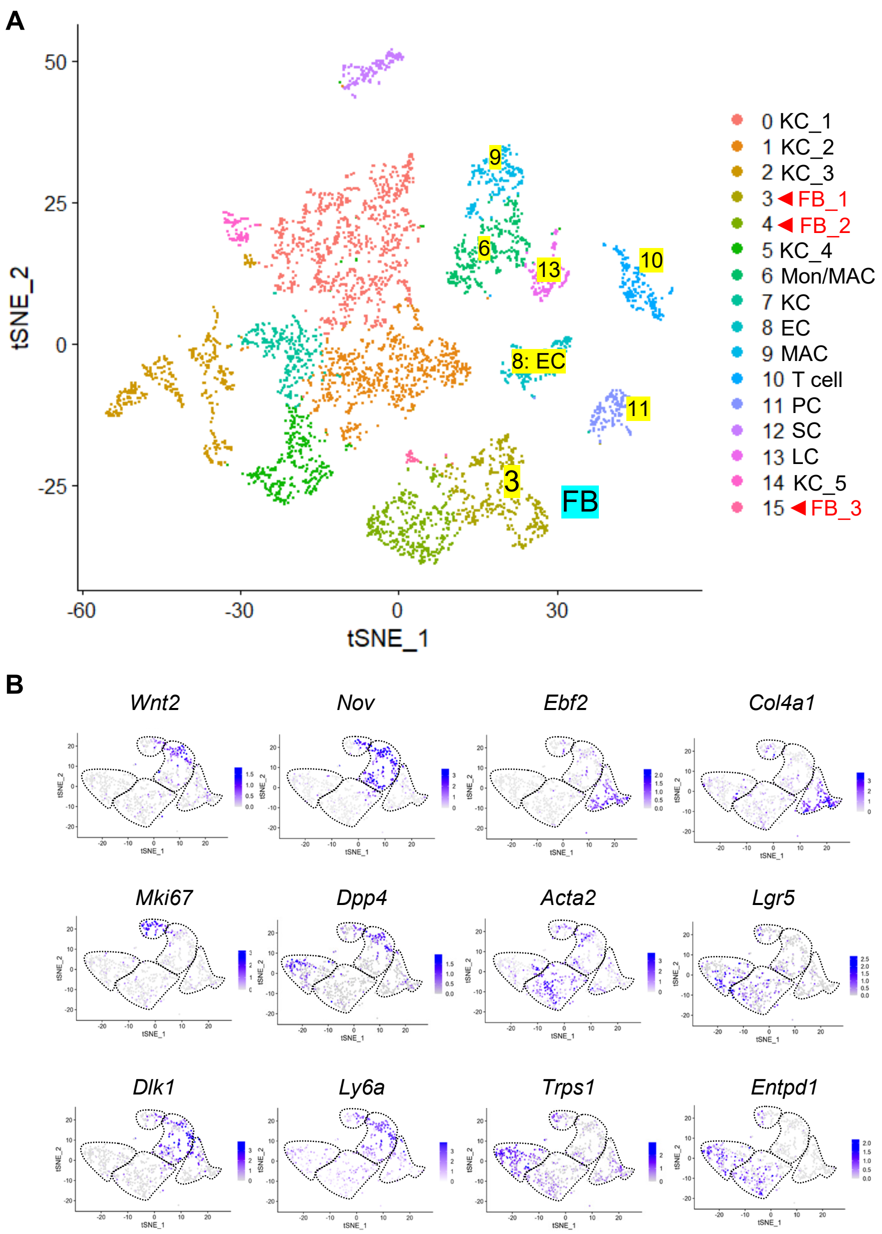

Figure S3. Single-cell RNAseq of the wound-induced center scar skin tissue
(**A**) tSNE plot of total wound single distributed by annotated unsupervised clustering. The identity of each cluster is shown on the right panel. KC, keratinocytes; FB, fibroblasts; Mon, monocytes; MAC, macrophages; EC, endothelial cells; PC, pericytes; SC, Schwann cells; LC, langerhans cells.
(**B**) tSNE plots showing the expression of marker genes on various *Pdgfra*$^{+}$ dFB sub-clusters.

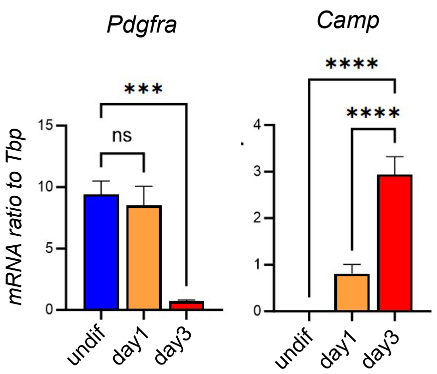

Figure S4. Analysis of gene expression during the in vitro adipogenic differentiation of dermal adipocyte progenitors
Primary dermal AP/pAds were treated with adipocyte differentiation cocktail for 3 days before cells were collected for qRT-PCR analysis of AP gene *Pdgfra* and early adipocyte marker gene *Camp* as indicated.

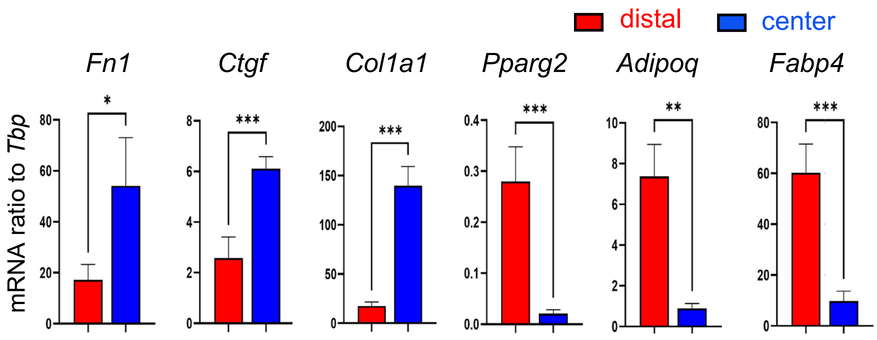

Figure S5. Activation of fibrogenesis and inhibition of dermal adipogenesis in the WISF model
qRT-PCR analysis of indicated fibrogenesis genes (*Fn1, Ctgf, Col1a1*) and adipogenesis genes (*Pparg2, Adipoq, Fabp4*) in wound center and distal skin samples.

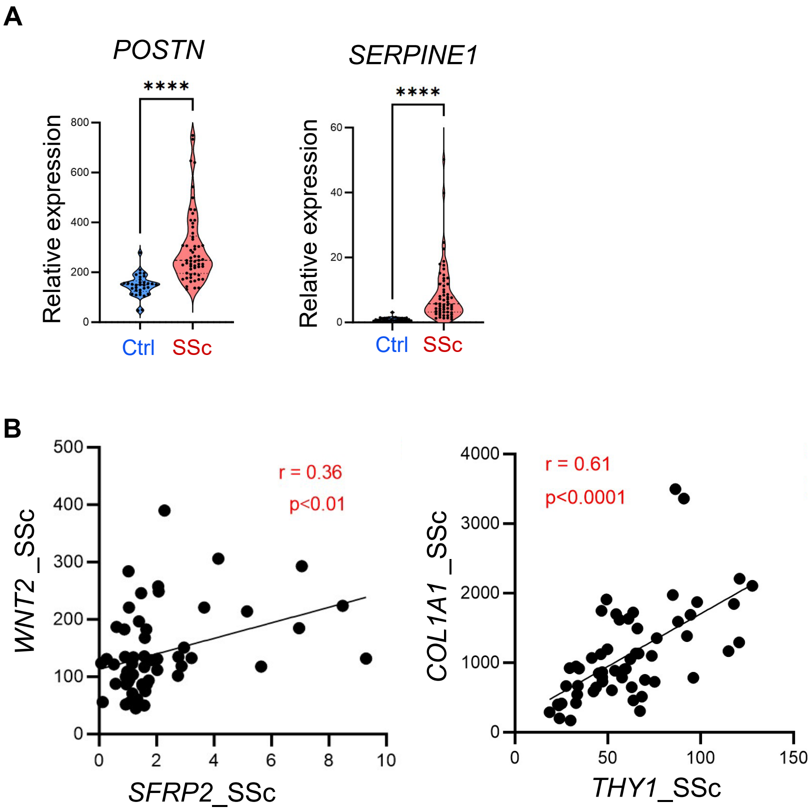

Figure S6. Analysis of gene expression related to AP and ECM in human scleroderma

(**A**) Violin plots showing the expression levels (Fragments Per Kilobase of transcript per Million mapped reads/FPKM values) of *POSTN* and *SERPINE1* in healthy controls or scleroderma (SSc) skin samples. All error bars indicate mean ± SEM. ****p< 0.0001.
(**B**) Correlation expression plots of indicated genes. Linear correlation analysis was performed by Pearson correlation coefficient method. The r value represents the correlation coefficent strength, and p value assesses the statistic significance of the correlation.
